# Adrenal crisis during pregnancy: Case report and obstetric perspective

**DOI:** 10.3389/fmed.2022.891101

**Published:** 2022-09-14

**Authors:** Barbara Gardella, Andrea Gritti, Annachiara Licia Scatigno, Anna Maria Clelia Gallotti, Francesca Perotti, Mattia Dominoni

**Affiliations:** ^1^Department of Clinical, Surgical, Diagnostic and Paediatric Sciences, University of Pavia, Pavia, Italy; ^2^Department Obstetrics and Gynecology, Fondazione IRCCS Policlinico San Matteo, Pavia, Italy; ^3^Institute of Radiology, Fondazione IRCCS Policlinico San Matteo, Pavia, Italy

**Keywords:** pregnancy, adrenal gland, adrenal insufficiency, adrenal crisis, obstetric

## Abstract

Adrenal dysfunction (AD) and, in particular, adrenal crisis are uncommon events in pregnant women, but associated with significant maternal and fetal morbidity and mortality if untreated or undiagnosed. Adrenal crisis may be confused with the common symptoms of pregnancy: the obstetricians should be able to promptly diagnose and treat it in order to avoid the adverse outcomes regarding the mother and the fetus. For this reason, AD must be treated by an expert multidisciplinary team. We presented a case report of a young pregnant woman with adrenal crisis due to tuberculosis, cocaine abuse, and massive bilateral hemorrhage with symptoms of emesis, hypotension, sudden abdominal pain, and leukocytosis. The most common issues of diagnosis and treatment are discussed and analyzed. Finally, we performed a review of the literature regarding adrenal crisis and adrenal insufficiency (AI) in pregnancy in order to clarify the management of these diseases in obstetrics setting.

## Introduction

During pregnancy, adrenal dysfunction (AD) and adrenal insufficiency (AI) appear as uncommon conditions, but they are associated with significant maternal and fetal morbidity and mortality if untreated or if undiagnosed during gestation or puerperium (1, 2). To understand the characteristics of AD during pregnancy and their obstetrical outcomes, it is important to consider the adrenal gland modifications during pregnancy. In particular, there is the development of hypercortisolism based on the modification of the hypothalamic pituitary adrenal axis, regulated by the feto-placental unit (3–7). During a physiological pregnancy, there is an increase in the values of total and free plasma cortisol, adrenocorticotrophic hormone (ACTH), corticotropin-releasing hormone (CRH), corticosteroid-binding globulin (CBG), and urinary-free cortisol (8–10). The level of cortisol increases from 11 weeks and reaches a peak around 26 weeks (4). The placental tissue is directly involved in the maternal pituitary and adrenal gland regulation. In this setting, estrogen causes an increase of CBG, while the placental tissue synthesizes CRH and ACTH, with an increase of the total cortisol level (11, 12). In particular, CBG increases 3–4-fold under the stimulation of estrogen, with an increase of the half-life of cortisol and total plasma cortisol values (3–7). Up to the 33rd week of gestation, maternal adrenal production provides for 90–95% of fetal cortisol synthesis. Subsequently, maternal adrenal function decreases in favor of fetal cortisol secretion (13–15).

In the last trimester, placental tissue affects the passage of fetal cortisol to the mother, while the placental 11-β hydroxysteroid dehydrogenase 2 has a protective effect on the fetus from excessive intake in case of excessive use of glucocorticoids (13, 16, 17).

The perturbation of the adrenal pathway results in a dysfunction of the adrenal gland. In particular, AI is characterized by decreased mineral corticoid and cortisol production due to reduced gland function (18). Approximately 44% of AI was reported as a primary disease, while 45% was reported as a secondary disease; moreover, hypothyroidism and Type 1 diabetes are often associated with AD (13). AI may present as an adrenal crisis (AC), or it can be chronic, known as Addison’s disease (18). Autoimmune atrophy of the adrenal gland represents 70–90% of cases of primary AI, with an estimated prevalence of around 110–140 per million people in the population and with an incidence of 4.7–6.2 per million people (19). The most common cause of primary AI is due to the autoimmune destruction of the adrenal cortex caused by autoantibodies against the steroid 21-hydroxylase enzyme. HLA-DRB1*04:04, a variant of the HLA-DRB gene, leads to an inappropriate immune response that leads to the autoimmune condition of Addison’s disease, although the pathological mechanism is currently unknown (20). Frequently, triggers such as viral infection can also lead to an autoimmune reaction. Pregnancy determines leukocyte activation with an increase of granulocytes and a decrease of lymphocytes and, as a consequence, the innate immune system increases its activity, while the adaptive immune system is downregulated. In the case of a triggering event, external antigens mimic sequences of self-antigens and activate T-cells through T-cell receptors and lead to an autoimmune process; this process is known as molecular mimicry (21).

Other causes that can lead to gland insufficiency include adrenal hemorrhage, adrenal infarction, cancer, illicit drug use, and infections. AI is diagnosed by the detection of low morning plasma cortisol levels with a diminished response to synthetic adrenocorticotropic hormone (ACTH) (18). A particular condition of secondary AI is Sheehan’s syndrome, caused by postpartum pituitary necrosis, and lymphocytic hypophysitis, which occurs in the postpartum setting or during the last trimester of gestation (13).

During pregnancy and puerperium the real incidence of AI is still unknown, and the literature data reported values ranging from 1 case in 3,000 pregnant women up to 5.6 per 100,000 and to 9.6 per 100,000 (8). Moreover, AI diagnosis is complicated due to the clinical presentation with abdominal pain, vomit, and shock, which could lead clinicians to suspect other pathological causes.

In addition, AC, especially during pregnancy, is a trigger for severe hypotension that may affect placental perfusion and therefore fetal wellbeing. This condition requires immediate care and treatment. The incidence of AC in pregnancy is reported in up to 69% of cases and it could potentially lead to primary or secondary AI. Severe hyperemesis and bilateral adrenal necrosis are the most frequent causes of secondary AI (13).

The diagnosis of AC is based on the presence of at least two of the following signs or symptoms: hypotension (systolic blood pressure < 100 mmHg), nausea, emesis, severe fatigue, hyponatremia, hypoglycemia, and hyperkalemia, which require parenteral glucocorticoid therapy (22). During pregnancy, this condition could be mistaken for acute abdomen, requiring surgery, and the most common condition of hyperemesis gravidarum. As we reported in Table 1, AC and hyperemesis gravidarum have the same clinical presentation; for this reason the table may be useful to identify the main finding of this condition for a successful diagnosis in clinical practice.

**TABLE 1:** Characteristics and management of AD in pregnancy and hyperemesis gravidarum.

Adrenal crisis
Know the physiological changes of adrenal axis during pregnancy Individuation of causes of adrenal crisis and management Monitor mother and fetus wellbeing Treatment of disease and prevention of complications *Clinical presentation* Abdominal and back pain Vomiting and nausea Shock Hypotension Severe fatigue Hyponatremia Hypoglycemia Hyperkalemia Pay attention to previous history of primary or secondary adrenal insufficiency *Clinical consideration* Blood samples for ACTH, cortisol, electrolytes, glucose Urine output examination Eventual fetal monitoring Fetal ultrasound evaluation Eventual RMI or CT scan evaluation *Management* Intravenous access Intravenous fluid replacement (0.9% saline solution or 5% dextrose-saline) in order to correct shock and hypovolemia Intravenous administration of corticosteroids for 2–3 days, and following corticosteroids replacement therapy *per os*. Identification of adrenal crisis cause (in pregnancy this could possibly mean the execution of the Synacthen stimulation test) Evaluation of fetal wellbeing: cardiotocography evaluation (gestational depending in accordance with obstetrics guidelines) and fetal ultrasound examination Delivery planning: vaginal delivery at term (obstetrics evaluation dependent)
**Hyperemesis gravidarum**
Know the characteristics of hyperemesis during pregnancy Individuation of nausea and/or vomiting causes Correction of Hypovolemia Reduce symptoms and prevention of complications *Clinical presentation* Persistent and severe nausea and vomiting requiring hospitalization Dehydration Electrolyte alteration Weight loss of more than 5% of pre-pregnancy weight *Clinical consideration* Patient’s medical history Weight monitoring Abdominal examination Urine output examination Plasma ketones and ketonuria identification Blood samples, electrolytes, glucose, urinary chemical, and microbiological examination *Management* Intravenous access Intravenous fluid replacement with appropriate electrolytes and vitamin supplementation Pyridoxine/doxylamine-pyridoxine or oral antiemetic’s Dietary changes advisement Fetal ultrasound examination

In the past, before the introduction of glucocorticoid replacement therapy, this condition was associated with an elevated incidence of maternal mortality (around 35–45%) (7, 23). Standard replacement therapy of AI is based on a daily regimen of multiple doses of hydrocortisone or cortisone acetate combined with fludrocortisone; the introduction of this treatment in clinical practice and the improvement of obstetric care and procedures for diagnosis have lead to a reduction in both maternal and fetal morbidity and mortality (7–13).

The replacement treatment is similar to that in non-pregnant patients: hydrocortisone is the most commonly used glucocorticoid for its pharmacological and pharmacokinetic characteristics, such as its short acting mechanism of action and its inability to cross into the placental tissue (2). This drug is used in 78.4% of cases alone or in combination with fludrocortisone (12, 23). In cases of AC, the first step is based on the evaluation of ACTH, cortisol, glucose, and serum electrolytes. The treatment provides intravenous saline infusion (2–3 l 0.9% saline or 5% dextrose in 0.9% saline) and intravenous Hydrocortisone treatment (100 mg every 6–8 h, or a continuous infusion of 200–300 mg in 24 h). In order to monitor the vital signs of the patient, serum electrolytes and urine output must be evaluated carefully (1, 3, 12). Intravenous hydrocortisone involves a treatment of 2–3 days and the subsequent transition to oral treatment with hydrocortisone and fludrocortisone (1, 3, 12). The regimen is based on two or three doses between 15 and 25 mg in order to mimic the physiological daily secretion of cortisol (12, 23). In pregnancies with inadequate compliance, prednisolone at a dose of 3–5 mg once daily could be acceptable (12, 23). During the treatment, an attentive evaluation of fetal wellbeing is mandatory with ultrasound examination and fetal monitoring in relation to the gestational period.

The hypothesized adverse effects of over-replacement therapy are the following: gestational diabetes, hypertension and pre-eclampsia, and weight gain and bruising, which are probably dose- and time- dependent (13). Experimental data reported that high doses of glucocorticoids may cause teratogenic effects, such as cleft palate, fetal death, and placental degeneration (24). On the other hand, the insufficient management could increase the risk of hyperemesis and the possible development of acute AC (13).

Endocrinological and radiological skills could permit a safe and fast approach for proper diagnosis. Literature data suggest the possibility to perform the Synacthen stimulation test during pregnancy and the repetition of this exam after delivery in order to confirm the diagnosis (1, 12, 25). In addition, the use of radiological imaging is possible and effective in pregnancy (after 16 gestational weeks), in AC, or in secondary AI (12, 13).

Finally, vaginal delivery is always the preferable choice, and only certain obstetric criteria can justify cesarean section (12). Obstetrical outcomes in patients with adequate and appropriate treatment of AI is good (2). Literature data reported obstetrical negative outcomes derived from AI: intrauterine growth restriction (FGR) is the most common reported event (4.2%). In addition, small for gestational age newborns were reported as a result of placental function failure (26). AI is associated with an increased number of cesarean deliveries and postpartum hemorrhaging (26).

It is important to highlight that pregnant women with untreated AI have an increased risk of adverse events which include preterm delivery, thromboembolism and acute AC, early miscarriage, and neonatal death (8, 2, 27–33).

Because of this, obstetricians should pay attention to this condition, especially considering the related symptoms such as nausea, emesis, fatigue, and altered food preferences which could be confused with pregnancy manifestations. Identifying AC in the absence of a previous history of AI, but in the presence of common symptoms, is a significant challenge in pregnancy. We introduce this case report in order to lead clinicians to a diagnostic pathway to clearly understand the clinical picture of AI.

## Case report

**FIGURE 1:**
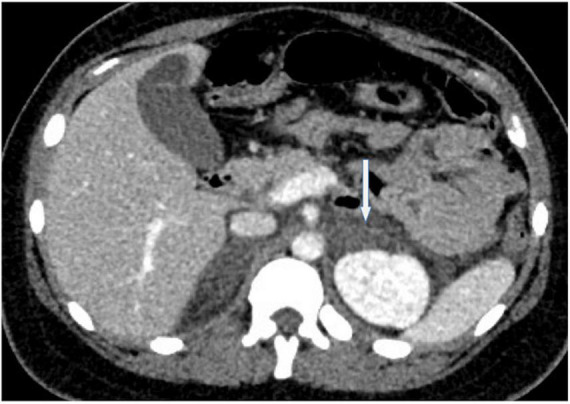
A representative image of adrenal glands with computed tomography. The axial un-enhanced computed tomography performed at time 0 shows enlarged and ill-defined adrenal glands and fluid film with a mean density of 50 Hounsfield Unit.

**FIGURE 2:**
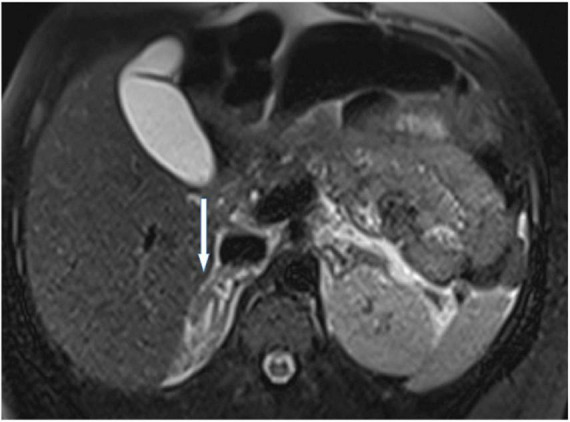
A representative image of adrenal glands with magnetic resonance imaging. T2 axial view at time 0 confirmed enlarged adrenal glands with hyperintense signal due to hyperacute hemorrhage. No focal lesions were described.

**FIGURE 3:**
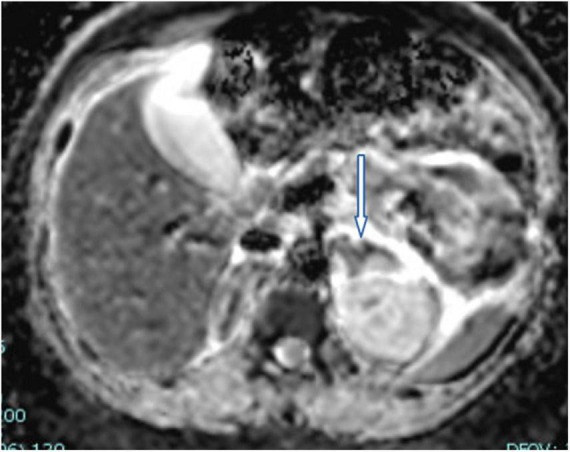
A representative image of adrenal glands with magnetic resonance imaging. The marked hypointensity signal confirmed the presence of adrenal hemorrhage.

A 26-years-old African primigravida patient was evaluated at our department at 30 + 2 weeks of gestation with abdominal pain and a reduction of uterine cervical length in a suspected preterm labor. During the observation, she developed a sudden onset of upper quadrant acute severe abdominal pain, which radiated posteriorly, complaining of nausea, emesis, and an increased state of agitation. In addition, severe hypotension was refractory to hydration. In order to exclude abrutio placentae or uterine rupture, we performed a cardiotocography and fetal ultrasound without detecting any abnormalities. The features of pain and the clinical findings could be associated with intestinal volvulus with vascular ischemia or acute pancreatitis. Blood exams showed an increase of inflammatory markers (WBC 31 × 10^3^/ml). Hemoglobin and pancreatic enzymes were in the normal range and urinalysis revealed no signs of blood loss or infection. For the worsening of pain, which was non-responsive to analgesics (paracetamol and opiates), she was scheduled for enhanced computed tomography (CT). It showed an imbibition of the adrenal lodges identifying ill-defined glands. The mean density was about 50 UH (Figure 1). With a hypothesis of adrenal disease, a magnetic resonance imaging (MRI) was performed and it confirmed the suspicion of hyperacute bilateral adrenal hemorrhagic infarction, associated with minimal left anterior pararenal blood fluid film without focal lesions (Figures 2, 3). The patient did not previously intake aspirin or heparin. ACTH was higher than normal (109 pg/ml) and Cortisol below the normal range (10.4 mcg/dL). Urinary metanephrines and normetanephrines were 5 times over the normal range (1461.2 mcg/24 h and 1,352 mcg/24 h, respectively), and urinary catecholamines demonstrated Dopamine 452.4 mcg/24 h, Adrenaline 123.7 mcg/24 h, and Noradrenaline 455 mcg/24 h. Electrolytes values revealed Hyponatremia, Hypomagnesemia, Hypocalcemia, and Hypokalemia: Na + 134.8 mEq/L; K + 3.14 mEq/L; Cl- 104 mEq/L; Ca + + 8.3 mg/dL, Mg + + 1.5 mg/dL. Hyperglycemia was reported: 185 mg/dL. The patient was transferred to the intensive care unit to permit intensive monitoring of her condition with suspected adrenal hemorrhage. During this period, fetal monitoring was performed to prevent fetal heart failure resulting from maternal hypotension. The treatment included intravenous saline infusion (2 l of 0.9% saline and intravenous hydrocortisone with a dosage of continuous infusion of 200 mg in 24 h).

**FIGURE 4:**
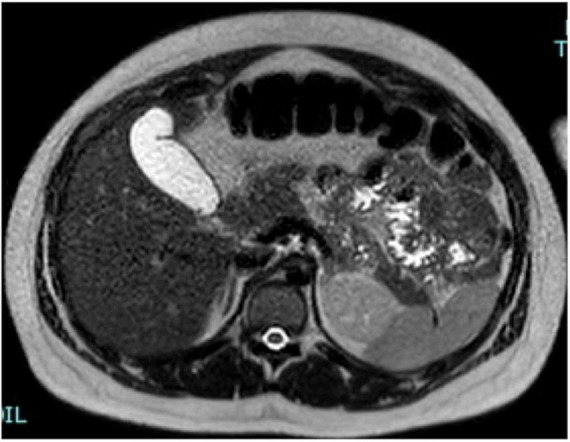
A representative image of adrenal glands with magnetic resonance imaging. After 4 weeks, the adrenal glands show normal shape, margins, and signal intensity in T2 axial view images. The film fluid was resolved.

After 3 days, the patient returned to the obstetric unit, and cortisone acetate was administered at a dose of 25 mg 1 + 1/2 tablet daily and antibiotic therapy was started upon the resolution of symptoms: Cortisol 6.9 mcg/dL, ACTH 38 pg/ml, normal plasmatic electrolytes, urinary normetanephrines 387 mcg/24 h, and metanephrines 866.6 h. Due to the nationality of the patient we decided to test for fecal and urine Koch’s Bacillus, which gave a positive result. The tuberculin skin test and the QuantiFERON test resulted positive. In addition, the patient tested positive for urinary cocaine metabolites because she had a previous history of drug addiction. The treatment in the hospital at the time of discharge after 4 weeks of hospitalization consisted of: cortisone acetate 25 1 + 1/2 tablet daily, rifampicin, ethambutol, and isoniazid. This therapy schedule was also continued after hospital discharge. The patient returned for a second MRI after 4 Weeks (Figure 4). The adrenal glands showed regular morphology and size, with substantially homogeneous intensity of signal.

The patient delivered a newborn weighing 2,795 g at term (40 weeks) Intravenous hydrocortisone was administered 100 mg iv during labor and followed by continuous infusion of hydrocortisone (200 mg in 24 h). No complications were seen during delivery. Post-partum was physiological with good therapeutic compliance. No medical issues have been identified in the child. Placental histology showed a massive macrophagous infarction of the membranes. No evidence of mycobacterium tuberculosis was retrieved in the specimens. The patient underwent regular follow-up which reported no secondary tuberculosis and showed good management of infection. The patient continued the endocrinological follow-up in another center.

## Discussion

Among the causes of AI and dysfunction during pregnancy, in our opinion, tuberculosis, cocaine abuse, and physiological hypertrophy of the adrenal glands contributed to massive bilateral hemorrhage. In addition, in pregnancy, clinical symptoms and signs such as weight loss, prolonged emesis, hyperpigmentation of the skin, hypoglycemia, hyponatremia, and hyperkalemia are the best predictors of AI (1, 12). For this reason, as we reported in Table 1, an accurate patient history and a correct differential diagnosis is the best indicator for the suspected diagnosis of AD.

As our case demonstrated, the pathway of adrenal hemorrhage may be identified in the amount of different pathological events and causes. Regarding this condition, literature data found three possible different mechanisms which were evolved in hemorrhage. First of all, one possible cause of adrenal deficiency and abdominal pain during pregnancy is adrenal infarction of a single vessel of the adrenal gland (31). Unilateral adrenal infarction is rare in pregnancy, but it is an important diagnosis to consider in order to prevent the incorrect management and worsening of the bilateral infarction. Glomski et al. identified 1.3% unilateral non-hemorrhagic adrenal infarction in pregnancy with acute abdominal or flank pain, detecting only one case of bilateral infarction. The identification of the MRI characteristics of this disease is important for preventing further invasive procedures or incorrect diagnoses (34).

Another cause of AI is spontaneous adrenal hemorrhage (SAH) which develops in the absence of trauma or adrenal tumor. The incidence of SAH is reported around 0.14–1.1% and it usually affects the right gland. During pregnancy, idiopathic unilateral SAH has rarely been described and the incidence is unknown (35). In case of bilateral involvement of adrenal glands, SAH may cause acute AC, shock, and death. In pregnancy, adrenal cortex hypertrophy and hyperplasia may lead to venous congestion and hemorrhage (36). Idiopathic adrenal hemorrhage usually causes acute AI and requires emergency adrenalectomy, and preterm delivery is proposed in hemodynamically unstable patients with massive hemorrhaging (37). For this reason, in our case, the patient was monitored in the Intensive Care Unit until her AC was resolved.

In our case, it is interesting that the patient tested positive to tuberculosis. Tuberculosis may lead to AI by direct glandular involvement, characterized by extra-adrenal infection, or by an indirect mechanism due to antituberculosis therapy. When primary AI is caused by direct glandular involvement, signs and symptoms may not appear until more than 90% of the gland has been damaged (38, 39). A review of the literature reported that in 566 patients affected by Addison’s disease with AI, tuberculous adrenalitis was observed in 70% of cases (39). Nevertheless, there is a geographical difference, because in countries where the incidence of tuberculosis has declined, the photogenic role of this condition in AI has decreased with an incidence of 10–15% of cases. On the other hand, the regions where the presence of *Mycobacterium tuberculosis* was significantly higher, the primary role of tuberculosis in Addison’s disease increased. In this case, primary AI resulted from the chronic involvement of the adrenal gland. Common clinical manifestations and symptoms may also appear for a long time after the initial involvement; only in a minority of cases is AI an isolated manifestation of early adrenal infection (5). In our opinion, this is a key-point, because our patient is African and the possible involvement of tuberculosis in our AI was promptly considered in order to ensure a correct diagnosis and therapy. A tuberculosis test must be performed in all pregnant women who refer pain radiating to the flank in order to exclude the diagnosis of adrenal tuberculosis, especially when they come from high-risk regions for infection.

Cocaine abuse, also in pregnancy, is associated with an alteration of the adrenal axis pathway; several literature data underlined the alteration of adrenal function and hormonal homeostasis derived from the cocaine user, not only in animals models, but also in human subjects (40). In pregnancy, the use of cocaine is also associated with adverse obstetrical outcomes such as preterm labor, congenital anomalies, FGR, abruptio placentae, low-birth-weight infants, neonatal death, development of pre-eclampsia-like syndrome, acute pulmonary edema, and cardiac arrhythmia (41). In addition, the intrauterine exposure to cocaine, in experimental models, revealed the downregulation of the fetal pituitary-adrenal axis, an increase of fetal ACTH, and the compromission in the cortisol response to stress, with a potential implication regarding neurodevelopment (42).

Literature data reported as the new diagnosis of AI is rare during pregnancy, because its diagnosis is generally performed prior to conception, with an adequate treatment in order to maintain adrenal homeostasis. Previous papers indicated a limited number of cases of AI (around 100 cases) recognized during pregnancy, mirroring the extremely uncommon condition (43). In addition, the development of AC represents a critical diagnosis, especially during pregnancy, because it requires a rapid empiric treatment in order to prevent adverse conditions, with an increase of mortality. Literature data reported a mortality rate in absence of adequate treatment that ranges between 35 and 45% (44). In a woman with a previous diagnosis of AD, the development during pregnancy of AC is an uncommon condition: the risk, based on literature data, ranges between 0.2 and 1.1%. On the other hand, the precise risk of AC in pregnancy is still unknown, in the absence of pre conception AD (2).

**FIGURE 5:**
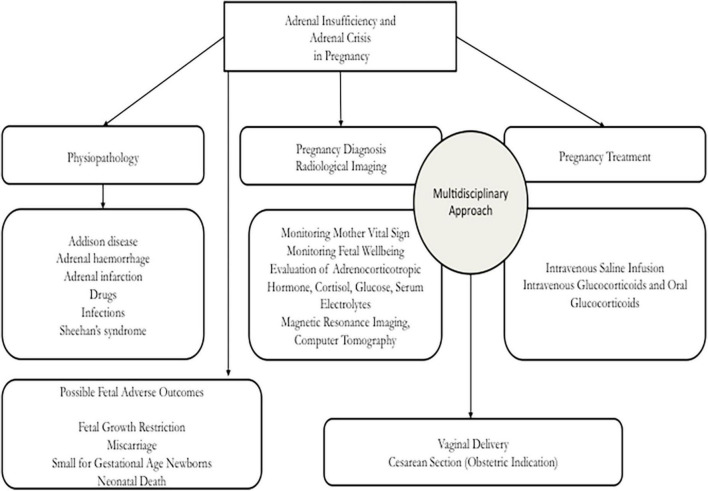
Flow-chart of management of adrenal crisis in pregnancy.

During pregnancy, it is important that adrenal deficiency is treated in an adequate center in order to guarantee appropriate resources and positive pregnancy outcomes to the mother and the fetus. On the other hand, it is necessary to underline that the diagnosis of the onset of AD is not a simple condition, because symptoms and indicators may be the only physiological modifications of pregnancy. For this reason, an expert evaluation is required in order to elaborate a correct diagnosis and management and to avoid inauspicious events for the mother and the fetus. In Figure 5 we presented a flow chart that may be useful to obstetricians in an emergency setting to identify the best diagnostic procedures in order to adequately manage the AC in pregnancy. In an obstetrics setting, the AI must be treated by an expert multidisciplinary team which is able to obtain a correct diagnosis, treatment, and positive results with the management of primary disease involved in the adrenal damage. Our analysis demonstrated that, during pregnancy, several pathological findings may fit the symptoms of back pain, vomit, and anomalies of blood value, but it is important not to ignore adrenal gland hemorrhage or tuberculosis. Nowadays, the rapid changes induced by pregnancy and the rapid migratory movements determine that certain pathologies are still very common. Our case, reported as the events analyzed in this paper, may be useful in an obstetrics setting to help other clinicians in the management of AI during pregnancy.

In addition, in our case, the patient, after the initial critical onset of diagnosis, reported with the prescribed treatment an electrolyte homeostasis and a hemodynamic stability during labor, delivery, and post-partum, supporting the fact that the immediate care and management favored good obstetrical and neonatal outcomes.

Regarding therapy of AI and AC, during pregnancy, literature data supported the evidence that the individualization of therapy in the third trimester of pregnancy does not have a stable certain schedule, but the dosage of glucocorticoids is personalized in each case (45).

For this reason, especially during pregnancy, it is important during the treatment plan to avoid the risk of over-treatment (for example, gestational diabetes or pre-eclampsia), and under-treatment (for example, electrolyte homeostasis alterations), in order to favor the stability of adrenal gland functionality and the adjustment of the therapeutic dosage, evaluating blood pressure and serum electrolytes as an indicator of treatment outcomes. Finally, also during delivery and post-partum, correct and personalized therapeutic management is necessary to prevent AC, basing each situation on a multidisciplinary consensus between obstetricians and endocrinologists, to obtain adequate outcomes.

## Conclusion

It is important that a multidisciplinary team manages the pregnant patient in order to prevent diagnostic delay and adverse events or an inadequate treatment of disease. Our aim is to provide an obstetric perspective in the management of AC in pregnancy.

## Data availability statement

The raw data supporting the conclusions of this article will be made available by the authors, without undue reservation.

## Ethics statement

Ethical review and approval was not required for the study on human participants in accordance with the local legislation and institutional requirements. Written informed consent from the patients/participants or patients/participants legal guardian/next of kin was not required to participate in this study in accordance with the national legislation and the institutional requirements.

## Author contributions

BG and AG wrote the manuscript. AS, FP, and AG were responsible for the collection of the clinical data of the patient. BG, AG, and MD designed and revised the manuscript. All authors contributed to the article and approved the submitted version.
